# A wake-up call: Sleep physiology and related translational discrepancies in studies of rapid-acting antidepressants

**DOI:** 10.1016/j.pneurobio.2021.102140

**Published:** 2021-08-14

**Authors:** Okko Alitalo, Roosa Saarreharju, Ioline D. Henter, Carlos A. Zarate, Samuel Kohtala, Tomi Rantamäki

**Affiliations:** a Laboratory of Neurotherapeutics, Drug Research Program, Division of Pharmacology and Pharmacotherapy, Faculty of Pharmacy, University of Helsinki, Finland; b SleepWell Research Program, Faculty of Medicine, University of Helsinki, Finland; c Experimental Therapeutics & Pathophysiology Branch, Intramural Research Program, NIMH-NIH, Bethesda, MD, USA; d Department of Psychiatry, Feil Family Brain and Mind Research Institute, Weill Cornell Medicine, New York, NY, USA

**Keywords:** Depression, Rapid-acting antidepressant, Sleep, Circadian rhythm, Pharmacology, Translational research

## Abstract

Depression is frequently associated with sleep problems, and clinical improvement often coincides with the normalization of sleep architecture and realignment of circadian rhythm. The effectiveness of treatments targeting sleep in depressed patients, such as sleep deprivation, further demonstrates the confluence of sleep and mood. Moreover, recent studies showing that the rapid-acting antidepressant ketamine influences processes related to sleep-wake neurobiology have led to novel hypotheses explaining rapid and sustained antidepressant effects. Despite the available evidence, studies addressing ketamine’s antidepressant effects have focused on pharmacology and often overlooked the role of physiology. To explore this discrepancy in research on rapid-acting antidepressants, we examined articles published between 2009–2019. A keyword search algorithm indicated that vast majority of the articles completely ignored sleep. Out of the 100 most frequently cited preclinical and clinical research papers, 89 % and 71 %, respectively, did not mention sleep at all. Furthermore, only a handful of these articles disclosed key experimental variables, such as the times of treatment administration or behavioral testing, let alone considered the potential association between these variables and experimental observations. Notably, in preclinical studies, treatments were preferentially administered during the inactive period, which is the polar opposite of clinical practice and research. We discuss the potential impact of this practice on the results in the field. Our hope is that this perspective will serve as a wake-up call to (re)-examine rapid-acting antidepressant effects with more appreciation for the role of sleep and chronobiology.

## 1. Introduction

The discovery of ketamine’s rapid antidepressant effects is arguably one of the most substantial advances in modern psychiatry. In contrast to conventional antidepressant drugs, which may require up to months of daily use before depressive symptoms are attenuated, a single infusion of subanesthetic-dose ketamine often relieves symptoms in mere hours (Berman et al., 2000; Diazgranados et al., 2010; Fava et al., 2020; Murrough et al., 2013; Zarate et al., 2012, 2006). Depressive symptoms are alleviated gradually, often reported to peak on the day following the treatment (aan het Rot et al., 2012; Berman et al., 2000; Coyle and Laws, 2015). However, ketamine’s antidepressant effects typically last for only up to a week, and some individuals do not achieve remission despite repeated administration (Phillips et al., 2019). These limitations have prompted unprecedented scientific inquiry into extending and reinforcing ketamine’s therapeutic effects or identifying an alternative, equally effective antidepressant agent that has fewer psychotomimetic effects.

Pharmacological, molecular, and functional effects associated with ketamine’s antidepressant effects have been extensively studied (Rantamäki and Kohtala, 2020; Zanos et al., 2018a). An emerging consensus highlights a central role of the stimulation of excitatory glutamatergic neurotransmission occurring primarily—although not exclusively—in the medial prefrontal cortex (Covington et al., 2010; Duman et al., 2016; Fogaça et al., 2020; Fuchikami et al., 2015; Hare et al., 2019; Kadriu et al., 2019; Kohtala et al., 2019; Kraus et al., 2019; Krystal et al., 2019, 2013; Rantamäki and Kohtala, 2020). Animal studies have identified numerous receptor-level changes and molecular pathways, many converging on mechanisms of synaptic plasticity and synaptogenesis, necessary for the rapid and sustained antidepressant effects of ketamine (Autry et al., 2011; Li et al., 2010b; Moda-Sava et al., 2019). However, the past two decades of drug discovery research have not been able to produce a single compound matching ketamine’s therapeutic efficacy; on the contrary, several promising drug candidates have failed in clinical trials (Garay et al., 2018; Kadriu et al., 2019).

The lack of translational success in the antidepressant field has traditionally been explained by poor construct validity of animal models of depression that are unable to capture the exclusively human aspects of the disorder (Cryan and Holmes, 2005; Harmer et al., 2010; Hasler et al., 2004; Nestler et al., 2002). However, it has become increasingly evident that many other physiological variables, such as selection of animal species and strain, biological sex and age of the animals, and even gut microbiota have a major impact on study outcomes and should be properly addressed in experimental design (Cryan and Holmes, 2005; Franklin and Ericsson, 2017; Võikar et al., 2001). Surprisingly, the effect of sleep—the most prominent neurophysiological change that occurs between ketamine treatment and the emergence of maximum treatment responses—has not received much attention. This work presents a perspective of why sleep and circadian rhythms may be the key to understanding ketamine’s antidepressant effects and how past research has broadly neglected these foundational physiological factors, which has potentially contributed to the soaring number of translational failures. We also discuss that this lack of attention to sleep and circadian rhythms may have prevented our ability to understand and exploit the full potential of ketamine and related rapid-acting interventions for the treatment of depression and several other neuropsychiatric disorders.

## 2. Sleep, depression, and rapid-acting antidepressants

Sleep is a universal phenomenon observed in virtually all animals throughout the phylogenetic tree (Cirelli and Tononi, 2008). It occupies a significant proportion of our lifespan, and its sustained loss is highly detrimental. The disconnection from external environment that coincides with sleep entails vulnerability to predators and impedes other activities important for survival, such as foraging for food. It can be argued that for such a costly process to survive through evolution, it must offer some other significant functional benefit. While the precise function of sleep remains enigmatic, it is known to play an important role in the maintenance of neuronal homeostasis and in the regulation of synaptic plasticity (Kuhn et al., 2016), learning (Yang et al., 2014), and memory (Tononi and Cirelli, 2014). Sleep restores energy supplies, promotes recovery from cellular stress, repairs cellular and DNA damage, clears metabolic byproducts, and regulates the immune system and temperature (Bass and Takahashi, 2010; Benington and Craig Heller, 1995; Inokawa et al., 2020; Mackiewicz et al., 2007; Refinetti and Menaker, 1992; Reimund, 1994; Zada et al., 2019). Most importantly, sleep quality is directly reflected in cognitive function and psychological health (Bonnet, 1985; Ford and Kamerow, 1989; Wong et al., 2013).

Depressive disorders are inherently associated with impaired sleep and circadian rhythm (Germain and Kupfer, 2008; Hasler et al., 2004; Norifumi et al., 2005; Nutt et al., 2008). Individuals with depression often exhibit decreased slow-wave sleep (SWS) (Ehlers et al., 1996) and lowered delta sleep ratio (Kupfer et al., 1990), which is the ratio of slow-wave activity (SWA; 0.5–4 Hz delta frequency) between the first two non-rapid-eye-movement (NREM) sleep episodes. Moreover, latency of rapid-eye-movement (REM) sleep and increase in total time spent in REM sleep are typical polysomnographic findings in individuals suffering from depression (Nutt et al., 2008; Pillai et al., 2011). Notably, classic antidepressant drugs produce varying effects on sleep (Winokur et al., 2001), suppression of REM sleep in particular, although the exact role of sleep in governing their antidepressant effects remains obscure. Problems in sleep and circadian regulation (e.g. early morning awakenings, altered melatonin and cortisol secretion) (Germain and Kupfer, 2008) often precede depressive episodes and may persist throughout remission, suggesting that their role in the pathogenesis of depression—at least in certain types—is primary rather than secondary (Li et al., 2013; McCarthy et al., 2019; Mishra et al., 2021). Indeed, emerging evidence has indicated that depressive symptoms can be alleviated by normalizing patients’ circadian misalignment and improving sleep quality (Ballesio et al., 2018; Cheng et al., 2019; Kalmbach et al., 2019; Manber et al., 2008; McCall et al., 2010).

Paradoxically, sleep deprivation—which entails keeping patients awake for prolonged periods of time—is known to induce rapid but highly transient antidepressant effects in a subset of depressed patients (Wirz-Justice and Van den Hoofdakker, 1999). Its antidepressant effects are suggested to emerge through the modulation of homeostatic or circadian processes of sleep (Bunney and Bunney, 2013; Orozco-Solis et al., 2017; Vogel et al., 1980; Wirz-Justice and Van den Hoofdakker, 1999; Wolf et al., 2016). Both sleep deprivation and ketamine regulate multiple molecular pathways involved in the neurobiology of sleep and share a similar circadian genomic signature in rodents (Duncan et al., 2013b, 2017; Duncan et al., 2018; Kohtala et al., 2020; Morgan, 2017; Orozco-Solis et al., 2017). Ketamine affects circadian motor activity patterns, reduce nighttime wakefulness, and lessen sleep disturbances, all of which correlate with better therapeutic response (Duncan et al., 2017, 2018; Vande Voort et al., 2017; Wang et al., 2021). Sleep deprivation leads to changes in well-known clock genes, which has been hypothesized to reset the dysfunctional clock gene machinery and stabilize circadian rhythmicity in depressed patients (Bunney and Bunney, 2013).

One of the most striking features coupling rapid-acting antidepressants to the neurobiology of sleep is the upregulation of SWA, which is thought to reflect the extent of synaptic potentiation preceding sleep (Rantamäki and Kohtala, 2020). Cortical excitation and synaptic potentiation are shared features of many interventions with rapid antidepressant potential, such as electroconvulsive therapy (ECT; Duman and Vaidya, 1998), transcranial magnetic stimulation (TMS; Baeken et al., 2013), and sleep deprivation (Vogel et al., 1980; Wehr et al., 1979; Wirz-Justice and Van den Hoofdakker, 1999). Several findings suggest that increased cortical activity—regardless of what triggers it—increase the buildup of homeostatic sleep pressure and lead to entrainment of circadian clockwork (Kohtala et al., 2020). Notably, postictal EEG slowing is one of the most frequently associated markers predicting positive therapeutic outcome of ECT (Farzan et al., 2014; Mayur, 2006; Nobler et al., 1993; Sackeim et al., 1996; Singh and Kar, 2017).

Rodent studies were the first to demonstrate the connection between ketamine and the upregulation of SWA in the early 1990s (Feinberg and Campbell, 1995). This association was revisited in a recent clinical study, where ketamine-induced amplification of SWA during the first NREM sleep episode on the first night after treatment was associated with clinical improvement in depressed patients (Duncan et al., 2013b). Another study has suggested that baseline delta sleep ratio is a useful predictor for the antidepressant effects of ketamine (Duncan et al., 2013a). These and other observations have led to hypotheses proposing that the interplay of increased synaptic strength and the neurobiological processes that occur during SWA is important for the emergence of rapid antidepressant effects as well as their sustainability (Duncan et al., 2019; Goldschmied and Gehrman, 2019; Rantamäki and Kohtala, 2020; Wolf et al., 2016). The clinical relevance of these ideas, however, remains to be thoroughly tested. Nevertheless, current evidence demonstrates that the time of day has an indisputable effect on neuronal function because cortical excitability increases with time spent awake (Huber et al., 2013) and also undergoes circadian fluctuations (Kuhn et al., 2016; Ly et al., 2016; Vyazovskiy et al., 2013). Therefore, considering that the key physiological effects invoked by rapid-acting treatments appear to converge on their capability to induce plasticity through transient cortical activation, it is also conceivable that the time of day influences their immediate and long-lasting actions in the brain.

Moreover, when designing experiments, it may be crucial to take into account the time of day during treatment because subanesthetic-dose ketamine is known to have excitatory and arousal-promoting effects (Abdallah et al., 2018; Li et al., 2016; Lu et al., 2008; Zanos et al., 2018a, b). These properties have been documented to delay and/or disturb sleep when the drug is administered during the inactive period in both preclinical (Ahnaou et al., 2017) and clinical settings (Irwin et al., 2013). It is tempting to speculate that some of the effects attributed to ketamine and other putative antidepressants in preclinical research may originate from unaccounted effects associated with the prolonged wakefulness of experimental animals resulting from the administration of stimulatory treatments in the early inactive period. This possibility further emphasizes the need to disclose the circadian time of treatment while reporting research in order to enable replication and proper comparison of results.

Nevertheless, the impact of sleep or circadian biology on drug effects in general is only rarely considered (Ruben et al., 2019) even though preclinical guidelines (ARRIVE) enforced by numerous publishers have included them among the essential details in reporting research during the past decade (Kilkenny et al., 2010; Percie du Sert et al., 2020). However, it should be noted that the most widely used clinical guidelines (CONSORT, SPIRIT, and TIDieR) do not clearly state the necessity of disclosing time of day and focus instead on other time-associated variables, such as treatment duration and frequency (Chan et al., 2013; Hoffmann et al., 2014; Schulz et al., 2010). These guidelines were initially issued to enhance the quality of research because it was found that important details that enable replication of clinical trials, including time-associated variables, were missing from half of the publications (Chan et al., 2013; Glasziou et al., 2008).

## 3. Overview of sleep physiology in studies of rapid-acting antidepressants

To the best of our knowledge, the impact of sleep or circadian time of drug administration has not been systematically evaluated in the context of treatment efficacy or potential translational bias in studies involving ketamine or other rapid-acting antidepressants. On the contrary, both sleep and chronobiology have received relatively little attention in the field. To illustrate this, we performed a literature search of English-language full-text original preclinical and clinical research articles about rapid-acting antidepressants published over the past decade (2009–2019) and archived in the Scopus database and examined the extent to which they considered sleep and circadian rhythm.

Scopus database was chosen over another commonly used database, Web of Science, because of its equal and more extensive coverage, respectively, of basic medical science and clinical medicine (Li et al., 2010a). The publication date range was selected because a steep increase in ketamine-related research began around 2008 (Rantamäki, 2019). The following query was used on August 7, 2020: (“rapid-acting antidepressant*” OR “rapid acting antidepressant*” OR “fast-acting antidepressant*” OR “rapid antidepressant*” OR “electroconvulsive therapy” OR “electroconvulsive shock” OR “ECS” OR “ECT” OR “ketamine”) AND “antidepressant”. The dataset was further restricted to preclinical (filtered by “animal” OR “animals”) and clinical studies (filtered by “human” OR “humans” OR “clinical”). Queries for preclinical and clinical papers resulted in 1218 and 2383 results, respectively, of which 1183 (97 %) and 2332 (98 %), respectively, were successfully sourced using open access and University of Helsinki library databases and analyzed using Alteryx Designer (Version 2020.3; Alteryx, Inc.; Irvine, CA, United States) and MATLAB (MATLAB 9.9; The MathWorks, Inc.; Natick, MA, United States) algorithm. Briefly, the script searched the availability of a paper from open access databases, and if failed, downloaded the article using Scopus API in accordance to its usage policy. The articles were downloaded as PDF files, optimized and converted to text using open source libraries (Tesseract OCR Engine, Poppler Tools), and imported to MATLAB array for a cleanup of non-text characters, hyphenated words, hyperlinks, and incorrect spellings. A count for the keyword “sleep” was subsequently performed using regular expression search, which was validated by a manual count of 50 randomly selected studies. Details of the articles used in analysis are presented in Table S1.

**Fig. 1.**
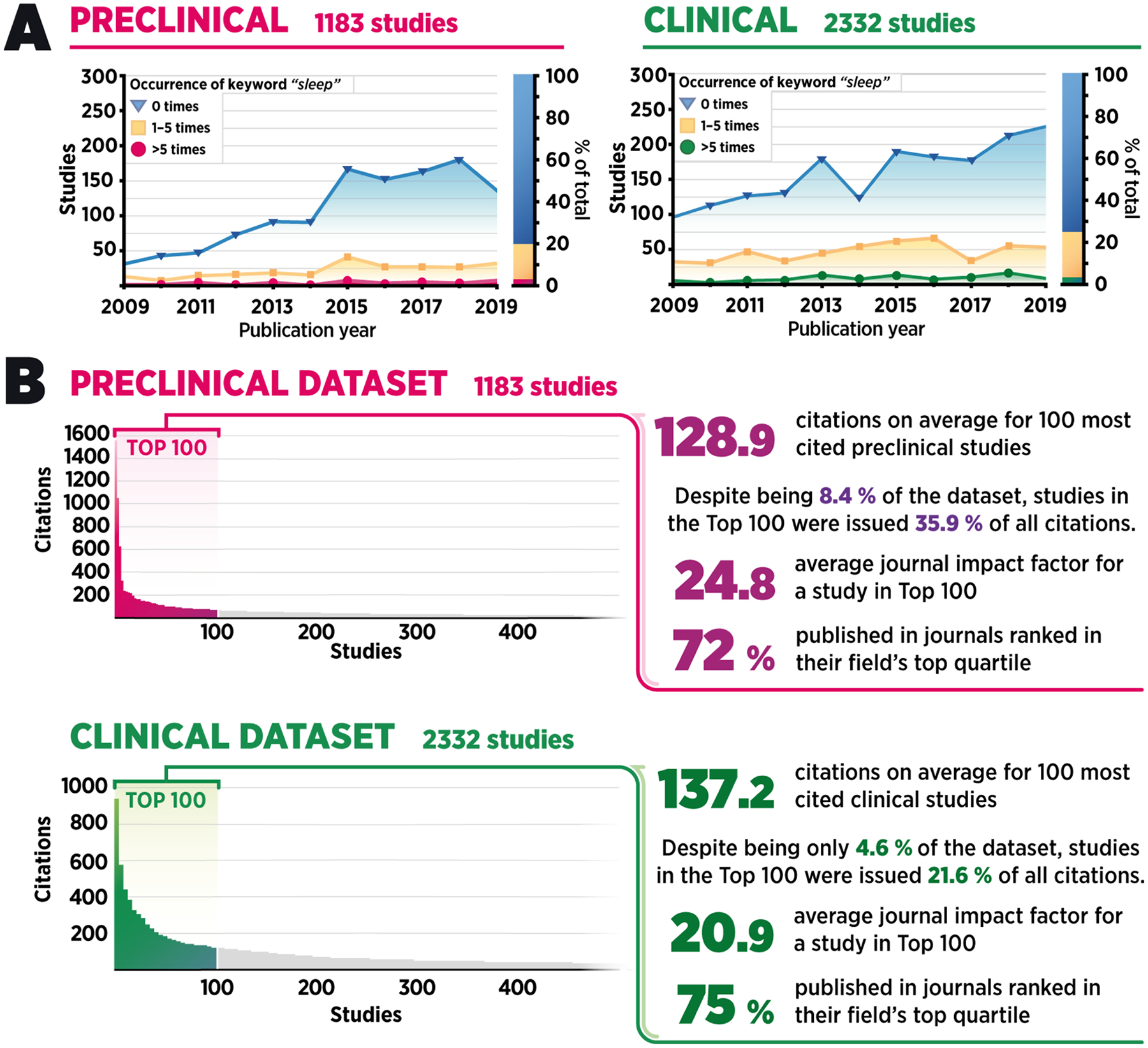
Mention of the keyword “sleep” in studies on rapid-acting antidepressants published between 2009 and 2019. **A**) A temporal overview of the whole dataset demonstrating counts for the keyword “sleep” in preclinical (n = 1183; left) and clinical (n = 2332; right) research papers. **B**) Citation count distribution in the dataset approximately follows the inverse-square power law—only a small fraction of all articles receives significant attention in the field.

Metadata-level information (e.g., abstract or author-supplied and generated keywords) available from Scopus indicated that sleep-related discussion was essentially non-existent in the entire dataset. Thus, to better understand the extent to which sleep is discussed in the study of rapid-acting antidepressants overall, we conducted a regular expression count for the keyword “sleep” in full-text articles, including references. A significant majority of papers included in the dataset (80 % and 75 % of preclinical and clinical papers, respectively) did not include the word “sleep” (Fig. 1). A fraction of studies (17 % and 21 % of preclinical and clinical papers, respectively) mentioned the word “sleep” between one to five times, and considerably fewer (3% and 4% of preclinical and clinical papers, respectively) did so six or more times. Notably, this pattern persisted throughout the publication period included in our study and did not correlate with number of citations or publication impact factor (data not shown), suggesting that sleep remains a widely unacknowledged topic in the field, especially in preclinical research.

**Fig. 2.**
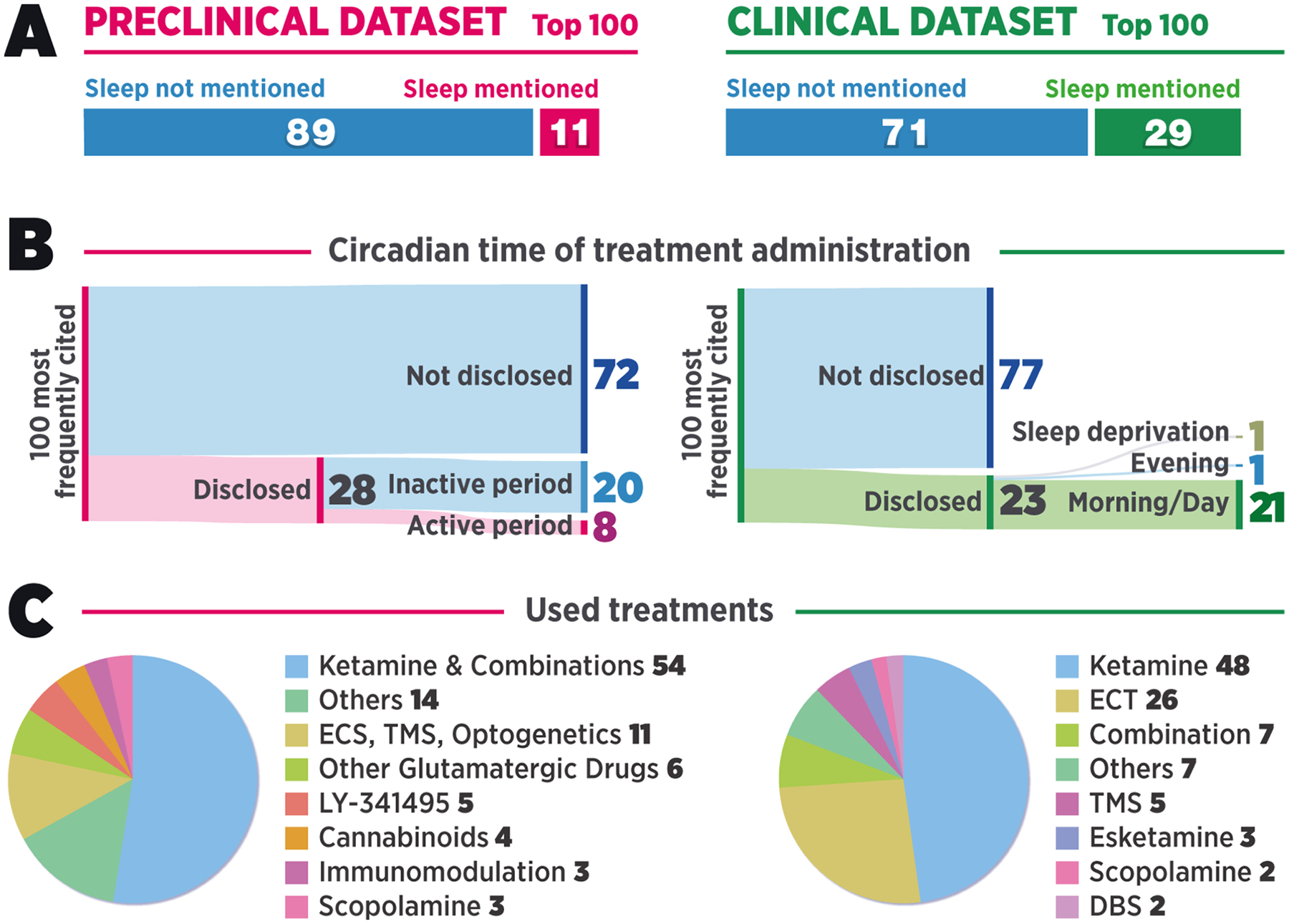
**A**) Proportion of the 100 most frequently cited preclinical and clinical research articles mentioning the word “sleep”. **B**) Disclosure of information regarding circadian time of treatment in the 100 most frequently cited preclinical (left) and clinical (right) research articles. **C**) The treatments used in the 100 most frequently cited preclinical and clinical studies. Abbreviations: ECS, electroconvulsive shock; TMS, transcranial magnetic stimulation; ECT, electroconvulsive therapy; DBS, deep brain stimulation.

Next, we performed a more detailed analysis of content, which was focused on the most cited research papers because they tend to receive the most attention within the scientific community, often guide future experiments, and act as the foundation for scientific theories. Thus, we selected the 100 most frequently cited original research articles involving a therapeutic intervention (i.e., reviews, case reports, and studies unrelated to antidepressant research were excluded) from both preclinical and clinical datasets for further manual analysis (Fig. 1). Notably, the vast majority of the publication forums belonged to high-ranked journals. The binary inclusion of the keyword “sleep” was searched from the main text, excluding references. Of these most frequently cited preclinical and clinical papers, only 11 % and 29 %, respectively, mentioned the word “sleep” more than once (Fig. 2), mirroring the results of the automated search. Furthermore, the few articles that did mention “sleep” typically did not discuss the role of sleep in the observed effects but rather mentioned it in the context of treatment timing (e.g., stating that the drug was administered “after overnight sleep at the clinic”) or in the introduction mentioning sleep impairments as one of the frequently observed symptoms in depressed patients.

Next, we investigated whether the observed lack of consideration for sleep was reflected in research practices of the 100 most frequently cited preclinical studies by analyzing them for the inclusion of details related to housing light cycles and the phases during which treatments were administered and behavioral testing was conducted. As expected, rodents (rats and/or mice) were the most widely used animal species in these studies, whereas two of the studies had been done in zebrafish. Notably, only eight papers reported administering the treatments during the animals’ active period (Fig. 2). Of these eight, two were conducted with day-active zebrafish (Riehl et al., 2011; Zakhary et al., 2011). Twenty of the animal studies were conducted during the inactive period, and most of the studies (72 %) did not clearly disclose the circadian time of treatment. It can be speculated, however, that a significant proportion—if not all—of the studies that omitted this information were conducted during the inactive period, as is customary in rodent research (Hawkins and Golledge, 2018).

Ninety-one of the preclinical studies employed behavioral analyses. Similar to circadian time of treatment, circadian time of behavioral testing was disclosed in only 35 studies, of which only seven had been conducted during the animals’ active period, whereas 20 had been conducted during the inactive period. Eight studies were counted as “mixed” because they either involved behavioral testing in both circadian phases or because the experiment times were only partially disclosed.

With regard to clinical practice and research, no existing treatment guidelines specify the optimal time for ketamine administration for the treatment of depression. Routine practice in the clinic is, somewhat obviously, to administer treatments during “office hours” that are roughly aligned with the early wake of the average human circadian cycle. Especially if fasting before treatment is required, treatments are typically administered as early in the morning as possible to reduce patient discomfort. While having an empty stomach is a stricter prerequisite for ECT, the medical agencies in both the United States and European Union also recommend fasting before administration of *S*-ketamine to avoid nausea, which is commonly experienced after treatment (European Medicines Agency, 2020; Daly et al., 2018; Janssen Pharmaceutical Companies, 2020; Popova et al., 2019). These practices were evident in our analysis of the 100 most frequently cited clinical articles; the disclosed treatment times were most often in the morning (19 studies), in the afternoon (one study; Baeken et al., 2013), or both in the morning and in the afternoon (one study; Ferrucci et al., 2009) (Fig. 2). Only one study stood out from the rest as it administered ketamine late in the evening (Irwin et al., 2013). In the experiment, terminally ill patients with comorbid depression received a nightly subanesthetic oral dose of ketamine that—perhaps unexpectedly—led to difficulty falling asleep as one of the reported adverse effects. One study involved overnight sleep deprivation as the treatment (Gorgulu and Caliyurt, 2009) and is therefore not directly comparable to the other protocols in terms of timing disclosure of circadian time of treatment. It should be noted, however, that only 23 out of the 100 clinical studies disclosed circadian time of treatment, mirroring the findings related to the preclinical literature. Regarding disclosure of circadian time of treatment, it should be noted that most of the timing-related information in clinical studies was phrased in a vague manner that left room for subjective interpretation (i.e., “before lunch” or “after overnight fast”). Additionally, it was apparent that the clinical studies were conducted during a significantly more homogeneous time range than the preclinical studies. Most experiments were aligned with early subjective wake of the subjects, and even the most ambiguous phrasing (e.g., “morning”) would generally be interpreted to span 5–6 h. In contrast, the circadian time of treatment in preclinical studies was distributed across both subjective preferred sleep and wake, and commonly reported ranges were as wide as 12 h (i.e., equal to the phase of a 12:12 h light cycle).

Taken together, the results of this perspective demonstrate an evident lack of consideration for sleep in experiment planning and reporting in studies investigating the effects of rapid-acting antidepressants. Admittedly, the employed method of analyzing the whole dataset using regular expression search was limited in nature—the search was limited to the keyword “sleep” without considering the use of its synonyms and inflections and included the use of the word in reference lists, and it may have missed some uses owing to optical character recognition. Nevertheless, the results indicate that sleep is overall a very marginal topic in the field of antidepressant research. Furthermore, the manual search of the most cited articles found that a similar proportion of papers mentioned the keyword, supporting the validity of the method. The fact that only a fifth of the studies in our dataset mentioned the word “sleep” demonstrates the existence of an implicit assumption that sleep and time of day are irrelevant to the outcomes of these experiments. This finding was further reinforced by our manual analysis of the top-cited preclinical and clinical studies, of which fewer than 30 % disclosed the experiment times. This oversight is surprising considering that many publishers have vowed to enforce reporting guidelines stating the necessity of disclosing experiment times. Our results demonstrate that these guidelines are not followed in practice, preventing the proper replication of studies and comparison of their results. The results also illustrate a substantial divergence between preclinical and clinical administration practices: while patients typically receive treatments during their early wake, experimental animals are commonly dosed during their resting phase.

## 4. Conclusion

The concepts highlighted by this perspective accentuate the complexity of current research on rapid-acting antidepressants. In the past decade, the 200 most frequently cited clinical and preclinical research reports—likely representing the most influential experiments in the field of antidepressant research—commonly ignored sleep and circadian physiology in experiment planning and reporting.

**Fig. 3.**
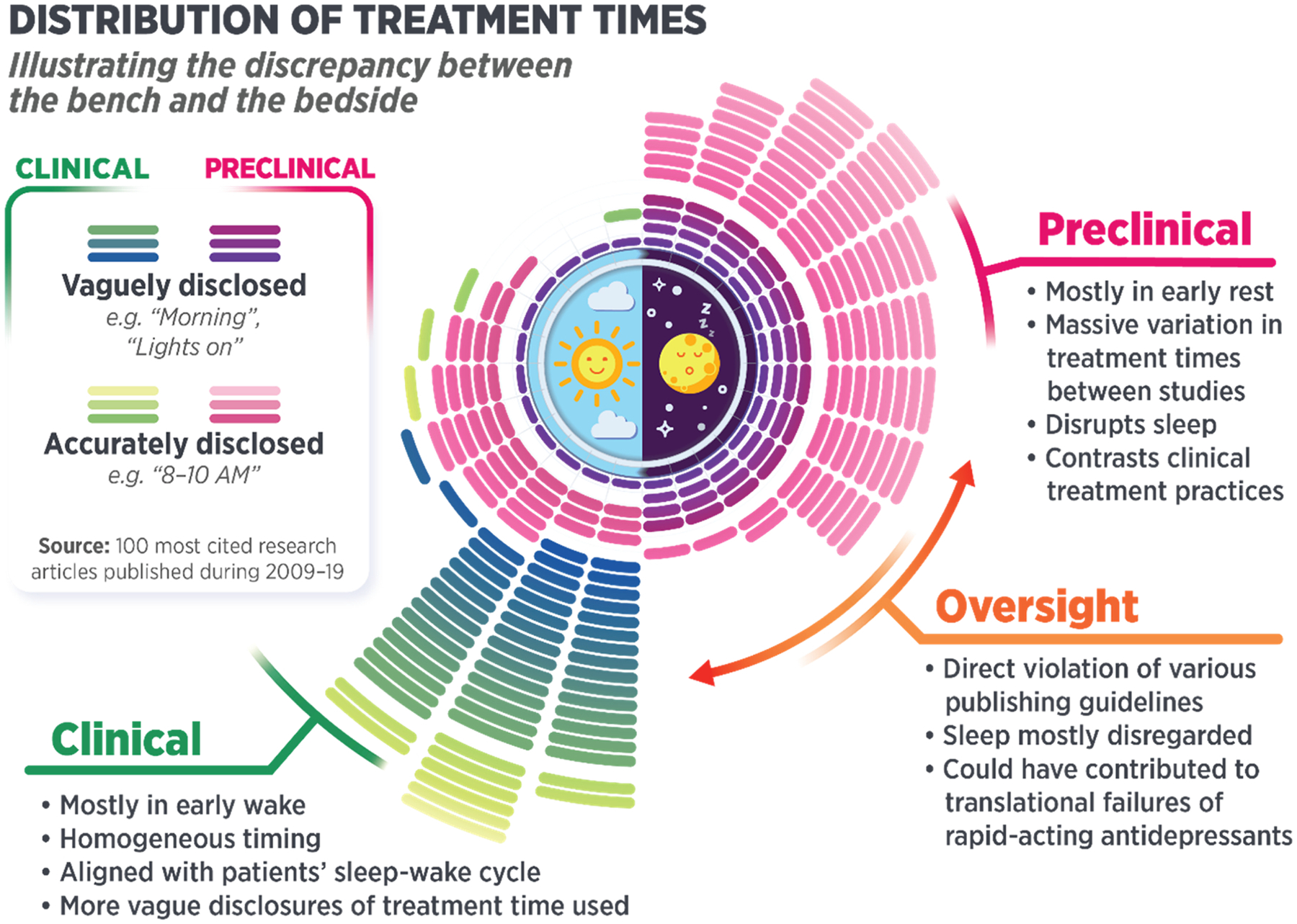
Illustration of the discrepancy between the bench and the bedside. Distribution of the times of treatment administration in the 100 most frequently cited preclinical and clinical studies on rapid-acting antidepressants.

One issue of particular concern is that the preclinical research studies that did disclose sleep-related variables and/or timing of experiment typically administered ketamine and other excitatory treatments during animals’ resting phase, potentially causing sleep disruption. This not only contradicts the regular sleep physiology of the animals but is also the polar opposite of clinical practice, in which patients are treated during their early waking hours (Fig. 3). Introducing such stimulatory treatments at night is likely to disrupt physiological sleep, which may influence or even account for experimental results. Indeed, it has been repeatedly shown that circadian disruption and incorrect circadian time of experiment induce significant variation in behavioral testing results (Cain et al., 2004; Chaudhury and Colwell, 2002; Karatsoreos et al., 2011; Verma et al., 2010) and in the effects of various pharmacological agents (Dallmann et al., 2014). This not only creates a clear divergence between preclinical research and standard clinical practice but also predisposes study results to a variety of unknown physiological and neurobiological sleep-related effects, such as increased synaptic potentiation due to a form of sleep deprivation.

Because this critical consideration has largely been omitted from the research literature—possibly owing to a lack of understanding of the important role sleep plays in physiological processes—it is conceivable that the sharp contrast in the features of preclinical and clinical research practices may have contributed to the soaring number of failed clinical trials of rapid-acting antidepressants. Another possible reason for the improper circadian time of experimentation appears to be simply that it is more convenient to conduct the experiments in well-lit environment. Indeed, while limited in number, both studies involving day-active zebrafish were conducted during the active period of the animals (Riehl et al., 2011; Zakhary et al., 2011), whereas experiments on nocturnal laboratory rodents were predominantly conducted during their inactive period. Even though the consequences of different circadian times of treatment on the effects of rapid-acting antidepressants require further research, we would argue that convenience is not an acceptable reason to introduce a significant source of variation to the results without at least properly addressing the practice while reporting research. In light of more recent hypotheses regarding antidepressant actions (Duncan et al., 2019; Morgan, 2017; Rantamäki and Kohtala, 2020; Zanos et al., 2018b), administering treatments during a more species-appropriate time would be an easy way to untangle the contribution of circadian disruption from the observed behavioral effects. We hope that the data presented in this report will encourage investigators to consider the effects of sleep (or lack thereof) in their experiments and clinical practice, and support research reproducibility by—at the very least—clearly disclosing circadian times for all experiments.

## Supplementary Material

Alitalo et al 2021-Supplementary Spreadsheet

## Abbreviations:

ECT: electroconvulsive therapy
NREM: non-rapid-eye-movement (sleep)
REM: rapid-eye-movement (sleep)
SWA: slow-wave activity
SWS: slow-wave sleep
TMS: transcranial magnetic stimulation
